# Accessing Artificial Intelligence for Clinical Decision-Making

**DOI:** 10.3389/fdgth.2021.645232

**Published:** 2021-06-25

**Authors:** Chris Giordano, Meghan Brennan, Basma Mohamed, Parisa Rashidi, François Modave, Patrick Tighe

**Affiliations:** ^1^Department of Anesthesiology, University of Florida College of Medicine, Gainesville, FL, United States; ^2^J. Clayton Pruitt Family Department of Biomedical Engineering, University of Florida, Gainesville, FL, United States; ^3^Department of Health Outcomes & Biomedical Informatics, University of Florida College of Medicine, Gainesville, FL, United States

**Keywords:** data curation, decision making, deep learning, artificial intelligence, electronic health record, machine learning

## Abstract

Advancements in computing and data from the near universal acceptance and implementation of electronic health records has been formative for the growth of personalized, automated, and immediate patient care models that were not previously possible. Artificial intelligence (AI) and its subfields of machine learning, reinforcement learning, and deep learning are well-suited to deal with such data. The authors in this paper review current applications of AI in clinical medicine and discuss the most likely future contributions that AI will provide to the healthcare industry. For instance, in response to the need to risk stratify patients, appropriately cultivated and curated data can assist decision-makers in stratifying preoperative patients into risk categories, as well as categorizing the severity of ailments and health for non-operative patients admitted to hospitals. Previous overt, traditional vital signs and laboratory values that are used to signal alarms for an acutely decompensating patient may be replaced by continuously monitoring and updating AI tools that can pick up early imperceptible patterns predicting subtle health deterioration. Furthermore, AI may help overcome challenges with multiple outcome optimization limitations or sequential decision-making protocols that limit individualized patient care. Despite these tremendously helpful advancements, the data sets that AI models train on and develop have the potential for misapplication and thereby create concerns for application bias. Subsequently, the mechanisms governing this disruptive innovation must be understood by clinical decision-makers to prevent unnecessary harm. This need will force physicians to change their educational infrastructure to facilitate understanding AI platforms, modeling, and limitations to best acclimate practice in the age of AI. By performing a thorough narrative review, this paper examines these specific AI applications, limitations, and requisites while reviewing a few examples of major data sets that are being cultivated and curated in the US.

## Introduction

Healthcare systems around the world have rapidly and pervasively adopted electronic health record (EHR) systems. Many countries report adoption rates higher than 90%, and the US is among this group with a reported 96% use as of 2017 (1–3). Currently, nearly 80% of all US office-based physicians have also adopted an EHR system to satisfy the specifications and requirements set forth by the US Department of Health and Human Services for such systems (4). The resulting underlying databases created by EHR systems contain large heterogeneous data sets that combine structured and formatted data elements such as diagnoses (International Classification of Diseases-10), procedures (Current Procedural Terminology® code), and medications (RxNorm), but also rich unstructured data such as clinical narratives, which represent over 80% of the data in EHRs (5).

Large healthcare systems realized the importance of this data early on and created data warehouses, now used both for research purposes and guiding evidence-based clinical practice. Such data warehouses not only contain EHR data, but also are often enriched with claims data, imaging data, “omics”-type data (e.g., genetic variants associated with a disease or a specific drug response), patient-generated data such as patient-reported outcomes (Patient-Reported Outcomes Measurement Information System®) (6) and wearable-generated data (e.g., nutrition, at-home vitals monitoring, physical activity status) from smartphones and watches. One example of the warehousing of large clinical data for research is the OneFlorida Clinical Research Consortium (7), funded by the Patient-Centered Outcomes Research Institute (PCORI). The OneFlorida Clinical Research Consortium is one of nine clinical data research networks funded by PCORI and aggregates, which harmonizes clinical data from 12 healthcare organizations that care for nearly 15 million Floridians in 22 hospitals and 914 clinical practices across all 67 counties of the state of Florida. This data repository functions alongside additional data warehouses that connect to larger systems that share healthcare data across different countries. The phenomenon of data sharing in healthcare is worldwide. For instance, the European Medical Information Framework (EMIF) contains EHR data from 14 countries, harmonized into a common data model to facilitate cohort discovery and research. With virtually unlimited capacity for data storage and advances in computational power for data analysis, the bottleneck is now in the development of appropriate methods to discover new knowledge to improve care.

Artificial intelligence (AI) methods, in particular machine learning (ML), reinforcement learning, and deep learning, are particularly well-suited to deal with both the data type and looming questions in healthcare. AI can aide physicians in the complex task of risk stratifying patients for interventions, identifying those most at risk of imminent decompensation, and evaluating multiple small outcomes to optimize overall patient outcomes. Integrating physicians into model development and educating physicians in this field will be the next paradigm shift in medical education. For example, the complexity of AI methodologies varies greatly, in turn impacting the ease of physician understanding and interpretation of results. Physicians frequently use decision trees as tools; however, they are effectively tied to the initial tree structure and thus somewhat static (8). On the other hand, deep learning models such as convolution neural networks are less easily interpretable, and may make it more difficult to establish a causal link (9); thus, the development of such models requires the active involvement of clinicians (10). Neural networks commonly used to decipher images collected from patients coupled with the corresponding interpretations often require involvement from radiologists to curate appropriate imaging data for training (11). A priori discussions by AI developers and medically informed physicians are necessary to define the levels of accuracy and interpretability that are required in each clinical context.

Despite methodological, societal, and ethical concerns (12), big data methods are being broadly adopted in healthcare systems for evidence-based clinical decision-making. In this paper, we discuss some of the major opportunities for how AI can assist healthcare workers in clinical decision-making. To prepare for this disruptive innovation, certain facets of medicine will be impacted earlier and more substantially than others. In this paper, we performed an narrative review of specific aspects of healthcare that we predict will most likely be first impacted by AI and how that impact can influence everyday clinical practice. Furthermore, this review includes the potential risks incurred by adopting AI as well as the requisite educational curricula changes and knowledge base needed to avert biases and prevent unsound decision-making.

## Methodology

We performed a comprehensive literature search using the databases PubMed, EMBASE, and Cochrane Review using the keywords (including alternative keywords): artificial intelligence, machine learning, deep learning, perioperative medicine, perioperative clinical decision making, preoperative risk stratification, machine learning and multi-objective optimization, machine learning and warning, machine learning and bias, and machine learning in medical education. Literature search included articles published between 2010 and 2020. Inclusion criteria were articles that focused on adult surgical patients, randomized controlled trials, observational studies, review articles, systematic reviews, and meta-analyses. Exclusion criteria were articles that focused on non-surgical encounters, editorials, letters to the editors, commentaries, books and book chapters, conference proceeding, and pediatric surgical patients. The scope of this review is perioperative clinical decision-making, including settings in the intensive care unit. In addition, we highlight the impact of AI on the future of medical education.

## Results

**Figure 1:**
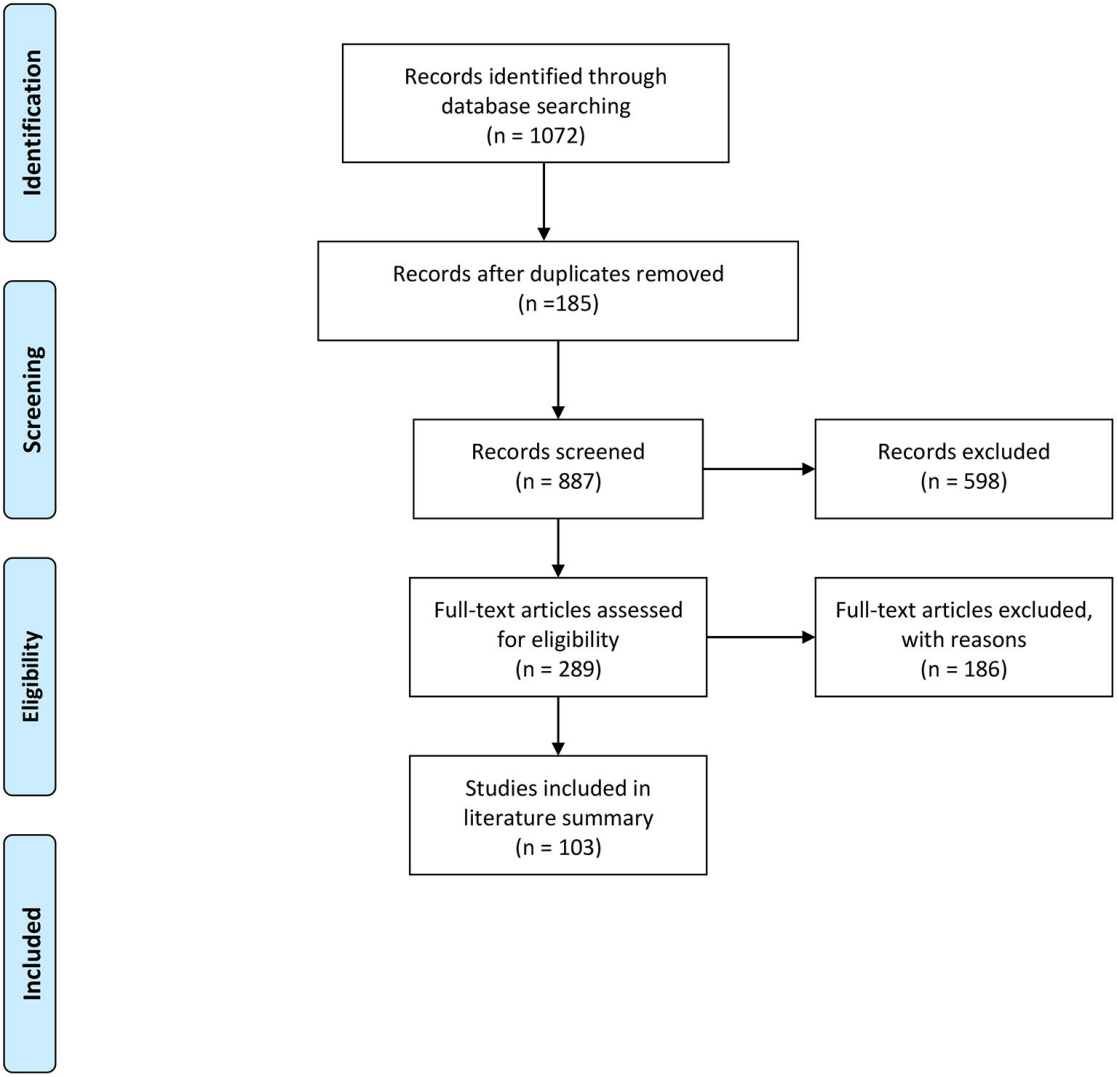
PRISMA flow diagram of accessing artificial intelligence for clinical decision-making.

An overview of the study's methodology and results is presented in Figure 1. The literature search yielded 1,072 abstracts, of which 185 were duplicates. The authors screened 887 abstracts and 589 were excluded based on the above exclusion criteria. The authors reviewed 289 full articles for eligibility and 186 articles were excluded because they did not meet inclusion criteria. The literature summary focused on 103 full articles. Upon completion of the literature review, we found that there were five main themes related to the role of machine learning, artificial intelligence, and clinical decision-making. The ever-increasing applications of AI methods and tools have potential in nearly every aspect of the clinical decision-making process. In this review, the scope was narrowed to three main promising AI application areas, the potential risks of implementation, and the requisite need for additional education. Specifically, the areas of application include: (1) risk stratification, (2) patient outcome optimization, (3) early warning of acute decompensation, (4) potential bias in ML, and (5) future medical training. These five areas were chosen based on consensus among the authors, who are familiar with recent literature and currently work and research within the AI space. Additionally, these areas reflect contemporary discussion points among clinicians, scientists, engineers, and policymakers given the continued public health burdens of acute illness, as well as the readily available detailed time series data for many at-risk patients. For a more detailed and granular review of AI and deep learning application, which is outside the aims of this review, please see (13, 14).

## Discussion

### Risk Stratification

ML models that can risk-stratify patients in preparation for surgery will help clinicians identify high-risk patients and optimize resource use and perioperative decisions. ML and AI can help clinicians, patients, and their families efficiently process all available data to generate informed, evidence-based recommendations and participate in shared decision-making to identify the best course of action. ML algorithms can be incorporated into several areas across the spectrum of care, either for disease management or in perioperative settings (15). Risk-prediction models have been used in healthcare practice to identify high-risk patients and to make appropriate subsequent clinical decisions. Appropriate risk stratification should result in proper resource use in this era of value-based care. Most risk-prediction tools are historically built based upon statistical regression models. Examples include the Framingham risk score, QRISK3 (for coronary heart disease, ischemic stroke, and transient ischemic attack), and National Surgical Quality Improvement Program (NSQIP). Unfortunately, many of these risk stratification methods are either non-specific and lack patient-level precision or require trained clinicians to review the records and specifically assess the risk. Healthcare systems have increasingly sought to use ML to assist in risk stratification, and these ML models may outperform statistical models in calibration and discrimination. A growing nationwide effort is seeking to enhance preoperative and perioperative support for high-risk patients and high-cost populations (16, 17). Preoperative evaluation clinics focusing on evaluating high-risk patients have shown improvement in 30-day postoperative outcomes (18). However, identifying these patients is challenging because of the difficulty in timely access to patient data coupled with the lack of robust predictive models. Many traditionally used models have been created to predict postoperative complications but with limited applicability at an individual patient level. Any predictive risk score is dependent on the underlying data and the technology used to process the data. In order to create a better prediction, high-quality, continuous data from multiple domains are required. Also, advancements in health data processing, biosensors, genomics, and proteomics will help provide a complete set of data that will enable perioperative intelligence (19). Furthermore, risk stratification is not limited to the preoperative setting. Incorporating intraoperative data for early detection of complications or clinical aberrations could also prevent inflammatory reactions that exacerbate the injury or high-risk interventions that may lead to iatrogenic injuries. Therefore, clinicians can use ML technology to build proactive systems to avoid these potentially destructive processes.

Multiple ML models that risk-stratify patients with a disease or prepare patients for surgery have been recently developed and validated (16, 20–25). These ML models have been shown to better predict mortality than conventional logistic regression after liver cancer surgery, aortic aneurysm surgery, and cardiac surgery. Other ML models have also been developed and validated to predict the risk of super-utilization and plan accordingly, starting in the preoperative setting in an increased effort to enhance value-based care (17). ML models to predict perioperative risk need to be accurate, locally calibrated, and clinically accessible. Changes in patient condition throughout the perioperative period can be included to update the risk assessment. The advantage of ML models in risk prediction is its automation capability, which is less burdensome compared to current tools (e.g., NSQIP). ML models allow for continuous recalculation of risk longitudinally over time, which can act as early-warning systems alerting clinicians to sudden changes. Incorporation of intraoperative data and interventions, such as hypotension, enable further interventions that enhanced recovery after surgery pathways emphasize. Another advantage is the promise that the use of ML in medicine will facilitate an understanding of what features drive outcomes (26). In perioperative medicine, ML can maximize the benefits of technology to provide safe, timely, and affordable healthcare. The key is integration of all data-generating platforms throughout all phases of patient care with collaboration to identify risks, detect complications early, and offer timely treatment (19).

### Patient Outcome Optimization

Optimization for each or the multiple potential patient outcomes is vital to the clinical decision-making process and the ensuing patient care. Typically, the requisite optimal steps, their timing, and the best sequence are determined by healthcare providers in consultation with family members. Despite best intentions, such decisions occasionally lead to suboptimal care due to the complexity of patient care, the increasing responsibilities of healthcare providers, or simply because of human error. The clinical decision-making process is often strictly based on standard guidelines and protocols that satisfy safety and accountability requirements. However, deviation from established protocols in complex care environments can be beneficial for the patient to adapt treatments for a more personalized regimen. In such dynamic settings, ML methods can be valuable tools for optimizing patient care outcomes in a data-driven manner, especially in acute care settings. ML and modern deep-learning techniques typically optimize an objective function (e.g., medication dosage) based on complex and multidimensional data (e.g., patient medical history extracted from EHRs). ML tools for optimizing care outcomes have been used in various settings, including critical care for optimizing sepsis management (27), management of chronic conditions (28), and optimizing surgical outcomes (29). Optimizing patient outcomes can be based on relatively simple yet efficient tools, such as decision trees in conjunction with the domain expertise to systematically codify accepted understanding of disease models and common treatments for patients. Although helpful in assisting with single-step decisions, these tools fail to consider the importance of sequential decision-making, which include many decisions that are dependent on previous actions.

Another more sophisticated approach is to use sequential decision-making tools that draw inspiration from related fields, such as operation research. For example, deep reinforcement learning models (30, 31) are based on well-known concepts such as the Markov decision process (MDP) (32) and Q-learning (33) adapted to neural networks. Reinforcement learning models learn to identify optimal policies based on a reward function. The policies are defined as a series of actions that culminate in the greatest reward, hence identifying the optimal policy. Recently, reinforcement learning and deep reinforcement learning have been used in several clinical settings, including optimal dosing and choice of medications, optimal timing of interventions, and optimal individual target laboratory values (34). For example, Nemati et al. used deep reinforcement learning to optimize medication dosing (35), and Prasad et al. (36) used a reinforcement learning approach to weaning mechanical ventilation in the intensive care unit. Although such tools hold great potential in optimizing the patient care process, safety and accountability is paramount. This could be complicated by the black-box nature of modern deep-learning approaches. The resulting policies may be dynamic and personalized, but their rationale may be challenging to interpret and explain. Additionally, unlike typical simulation and gaming environments, applying reinforcement learning in clinical settings is much more challenging. It is not trivial to identify the most suitable reward structure, and the effects of treatments can be non-deterministic. In such settings, it is difficult to solve the credit assignment problem, i.e., to demonstrate that deviations from the protocol based on a reinforcement learning suggestion were beneficial for the patient. Future approaches also could examine different time scales. For example, although early interventions (e.g., early antibiotics) may not lead to immediate improvements, they could culminate in the greatest ultimate reward (e.g., higher survival rate).

Patient outcome optimization such as reinforcement learning methods can ultimately provide a tool to help standardize care at health systems of different scales. This could provide a more equitable healthcare system, especially in rural and remote settings.

### Early Warning of Acute Decompensation

Acute decompensation is uncommon, but it is typically accompanied by increasing physiologic derangements and worse outcomes. Intervening early may mitigate poor outcomes; however, it is often difficult to identify this patient population before significant hemodynamic compromise with our traditional standard monitoring and commonly used early-warning scores. Six to eight hours may precede such acute patient decompensation, which can easily provide ample time for interventions to be made (37). The EHR contains a large amount of data that may be useful to identify patients at the highest risk of decompensation if the data are evaluated over time (37, 38). Multivariate regression-based models or AI-based early-warning systems have the potential to detect subtle trends in physiologic parameters over time to provide precision and reliability (38–41).

Vital sign monitoring and associated alarms were one of the earliest methods to detect patient decompensation (40). They are effective in alerting providers to discrete vital sign abnormalities in real time; however, early or isolated vital sign abnormalities also may fail to signal to providers an impending decompensation (40). Once it becomes evident that a patient is decompensating, the initial response is often directed toward correcting one or more abnormalities until an etiology is determined. The Modified Early Warning Score (MEWS), Rothman Index, Sequential Organ Failure Assessment Score (SOFA), and quick SOFA (qSOFA) were developed to incorporate multiple vital sign abnormalities to identify at-risk patients before decompensation occurs. The drawbacks to these scores are that even if they are automated and incorporated into the medical record, they rely on discrete data points of pre-existing vital sign changes and are subject to reporting error. Additionally, because of their high sensitivity but low discriminatory ability, these scores identify a large number of patients as “at risk” when the actual number is far lower (38). Furthermore, because interventions often involve their own risks, they may not be implemented until it becomes clear that a patient's condition is rapidly deteriorating. At that point, immediate and possibly emergent interventions that are themselves high risk and invasive must be performed. Preventative measures may be taken earlier and with more accuracy if AI metrics are implemented as opposed to the traditional risk-evaluation scores (40, 42). AI-based monitoring incorporated into the EHR can facilitate the use of large volumes of data for all patients more efficiently and precisely than a physician could, enabling AI to identify patients who are most at risk.

The operating room may be one of the most challenging areas for early detection, workup, and treatment of acute decompensation. The Hypotension Prediction Index (HPI; Edwards Lifesciences, Irvine, CA) is an algorithm created to aid in the early detection of intraoperative hypotension, defined as mean arterial pressure <65 mmHg for non-cardiac surgeries (41, 43, 44). It is now incorporated into the Edwards monitoring system. It was developed using an ML, logistic regression-based model analyzing components of the arterial waveform (41, 43, 44). One advantage is that in addition to early notification of hypotension, this tool also identifies some of the most likely causes for the predicted hypotensive event, e.g., vasoplegia, hypovolemia, or possibly conditions related to cardiac contractility. Initial studies, although small, single center, and not without bias, indicate that the HPI and implementation of the monitor were reasonably effective in preventing clinically significant hypotensive events. Although developed with AI, this monitor and associated alarm rely on the data that it was trained and developed on, and they do not learn and adapt with each patient.

Using AI to effectively create an early-warning score using time series data from the EHR presents many challenges. An ideal score would identify patients before an obvious decompensation. It would have excellent discriminatory ability so that physicians would have confidence implementing appropriate interventions as well as transparency to identify the sources of risk and the reasons for decompensation. Incorporating appropriate treatments and their effects on risk reduction remains a weakness of all existing early-warning systems. AI-based algorithms using time series data from the EHR are in development with strong results. Shickel et al. used a modified recurrent neural network model on temporal intensive care unit data to develop deepSOFA, a real-time mortality risk prediction score based on the traditional SOFA score (38). Its predictive ability performed well in identifying increased risk of mortality. Lauritsen et al. developed the explainable AI early-warning score (xAI-EWS). It is meant to be incorporated into the EHR and uses a temporal convolutional network and deep Taylor explanation model to provide predictions. It has demonstrated feasibility using predictions for risk of acute injury, sepsis, and acute lung injury (39).

### Potential for Bias in ML

As AI becomes more pervasive in both public and personal health across diverse populations, there have been increasing concerns, and related examples, of AI solutions leading to inadvertent bias of modeling results (45–48). Broadly, such bias can originate from the data used for model training and testing, as well as the mechanics of the model itself (49). Bias originating from data can be pernicious; for instance, work by Weber et al. found that simply filtering for “complete” EHRs, a common strategy for managing missing data, introduced a bias toward older patients who were more likely female (50).

Less pernicious examples include reference imaging datasets in which more than 80% of subjects were light-skinned individuals (51). With respect to modeling mechanics, the non-linearities, extensive interactions among variables, and difficulties interpreting how ML models arrived at their results, ML also presents many new challenges to addressing sources of inadvertent bias that differ from classifiers that enforce linear models of independent variables in smaller, more manageable datasets. Under the rubric of decision support, an unfair algorithm has been defined as “one whose decisions are skewed toward a particular group of people” (49). Verma and Rubin have clarified several definitions of algorithmic fairness, where definitions are based on objective probabilistic assessments (52). These definitions help provide a platform for promoting algorithmic fairness by creating neutral models through approaches addressing anti-classification, classification parity, and model calibration on protected attributes (53). Notably, these solutions may present their own ethical issues. McCradden et al. (54) suggest that some solutions to algorithmic fairness can instead reinforce health inequities and even exacerbate harms to vulnerable groups. Until more robust solutions to the challenges of algorithmic fairness can be identified and implemented, physicians should remain vigilant for how ML models, built on training samples from general populations, may be misapplied to their own patients. This appreciation of ML building and application will require a new level of professional development and commensurate medical education curricula, which will be discussed in the next section.

### Paradigmatic Shift in Medical Training

Applying advances in biomedical informatics and ML models to patient care will require clinicians to reconsider their educational training and infrastructure. Wartman et al. noted that the practice of medicine is transitioning from the Age of Information to the Age of AI (55). Traditionally, medical curriculum has been founded on memorizing a massive curriculum, applying it to a learned clinical experience, and determining the validity of ensuing information as it becomes published. Similarly, understanding principles of normal variants of anatomy and physiology, followed by an examination of pathophysiologic variants, presents students with a model-based rubric in which to incorporate each new wave of information learned through personal experience as well as throughout the medical literature. This paradigm has also permitted physicians to extrapolate previous understanding by logic and experience to novel diagnostic reasoning and therapeutic approaches by extension of previous models. However, the amount of information has become insurmountable. The time for medical information to double was 50 years in 1950, 7 years in 1980, 3.5 years in 2010, and a staggering estimate of 73 days in 2020 (56). Humans are not only incapable of this level of exposure or retention, but the magnitude has also created substantial levels of stress-induced mental illness among learners (57). Fortunately, advances in biomedical informatics point to new approaches that can seamlessly synthesize old and new medical information. These advances will provide the foundation for AI advances to recognize patterns of patient information to help diagnose, treat, and manage patients. This transition will require the development of new knowledge, skills, and attitudes by healthcare workers. Furthermore, it will require a rethinking of the medical school curriculum, in which new data analytics methods are carefully integrated with traditional medical education. In an extremely busy curriculum and at a time of numerous other considerations, such as climate change (58), incorporating AI will present challenges.

Many of the AI subfields such as ML and deep learning use complex algorithms that generate outputs from seemingly opaque non-linear functions that most physicians likely find difficult to understand or incorporate into their existing approaches to evidence-based medicine. Subsequently, this black-box phenomena (10) will be difficult for physicians to trust, and it will also be a challenge for the doctor-patient relationship since many physicians will find themselves unable to explain the diagnosis, prediction, or therapy (59). This challenge will increase with the stakes and timeliness of the given issue; for instance, outcome assessments involving the withdrawal of care may pose heightened anxiety regardless of the model's accuracy. Therefore, physicians will need to develop a basic understanding of how input data are aggregated, analyzed, and generated into specific pathways of care for individualized patients. Furthermore, these algorithms will require physicians to have a better understanding of calculus and linear algebra, manipulation of data sets (curation, provenance, quality, integration, and governance), and model performance metrics fundamental in grading AI algorithmic decision-making. This knowledge will allow physicians to recognize when AI algorithms are being used on inappropriate patient populations, when AI tools have become outdated and need updating, or when aggregated data is biased. These new AI clinical decision-support systems have limitations in their application to patient populations, contextual changes, and therapeutic variances that will require a stronger appreciation of probabilities and confidence ratings (59). It will also be important to understand when physicians are justified in deviating from AI-inspired treatment protocols. Physicians will need to update their understanding of evidence-based medicine principles to include modern approaches to analyzing and assessing causality to ensure a robust understanding of how patients, social determinants of health, and healthcare systems interact to inform health-related outcomes. Physicians practicing in the age of AI should be competent in the effective integration and data use that emerges from an endless array of sources.

The emerging need for understanding how AI data platforms function and generate predictions is juxtaposed with the ever-important traditional need for communication skills, empathy, and teamwork. Translating the predictions from complex AI algorithms into meaningful and personalized information for patients will require strong communication skills as well as compassion. Compounding this resurgence of social skills requisition for medical practice will be the application of cognitive psychology principles. Understanding this need for social skills will help identify biases and heuristics that impact decision-making as well as help physicians frame choices, understand context, and have neutral but meaningful conversations with patients (55).

## Conclusions

The ubiquitous adoption of EHRs in healthcare systems around the world has created vast repositories of personalized data sets that are perfectly fitted for AI to examine, develop, and predict upon. The subfields of ML and deep learning networks have shown success in providing solutions to the healthcare questions of risk stratification and optimizing patient outcomes. Use of this technology will exponentially expand as it is increasingly integrated into large healthcare systems. AI capabilities will aide physicians in weighing competing healthcare goals and numerous risks by facilitating multiple outcome optimization of outcomes that are too difficult to recognize and navigate on an individual and isolated basis. Healthcare workers will be expected to comfortably work within this new AI frontier and in turn relate it to their patients. Furthermore, physicians must be able to interpret the predictions of these AI algorithms as well as deconstruct the models from which they ebb. In addition, physicians will need to recognize plausible bias and the appropriate patient population application that stems from understanding the training cohort used to create the model. This understanding will require additional medical education and professional development for current practitioners and a revamped curriculum for all new learners currently in medical school. Most importantly, physicians must maintain and cultivate emotional intelligence and compassion when relaying results and recommending interventions from these complex models to uncertain and vulnerable patients who want to make informed decisions for themselves or a family member's well-being.

## Author Contributions

CG, MB, BM, PR, FM, and PT contributed substantially to all aspects of the work, including conception, literature review, manuscript drafting, critical revision, and agreed to be accountable for all aspects of the work. All authors contributed to the article and approved the submitted version.

## Conflict of Interest

The authors declare that the research was conducted in the absence of any commercial or financial relationships that could be construed as a potential conflict of interest. The reviewer, TL, declared to the editor a past collaboration with the authors, and confirms the absence of ongoing collaborations at the time of the review.
